# Feet Salvage Using Anterolateral Thigh Flaps after Severe Frostbite Injury: A Case Report

**DOI:** 10.3390/jpm14040389

**Published:** 2024-04-05

**Authors:** Krešimir Martić, Borna Vojvodić, Božo Gorjanc, Ivan Budimir, Hrvoje Tucaković, Doroteja Caktaš, Rado Žic, Josip Jaman

**Affiliations:** 1 Department of Plastic, Reconstructive and Aesthetic Surgery, Dubrava University Hospital, Avenija Gojka Šuška 6, 10000 Zagreb, Croatia; kmartic@kbd.hr (K.M.); bgorjanc@kbd.hr (B.G.); ibudimir@kbd.hr (I.B.); htucakovic@kbd.hr (H.T.); dcaktas@kbd.hr (D.C.); rzic@kbd.hr (R.Ž.); jjaman@kbd.hr (J.J.); 2 Department of Surgery, School of Medicine, University of Zagreb, Šalata 2, 10000 Zagreb, Croatia; 3 Clinic for Traumatology, Sestre Milosrdnice University Hospital Center, Draškovićeva 19, 10000 Zagreb, Croatia

**Keywords:** frostbite, anterolateral thigh flap, feet salvage, reconstructive surgery

## Abstract

Background: Frostbite is a severe injury characterized by tissue damage due to exposure to freezing temperatures. It often necessitates prompt medical intervention to prevent further complications such as necrosis and amputation. This case report explores the successful use of bilateral anterolateral thigh (ALT) free flaps for feet salvage in a 19-year-old male refugee from Gambia who suffered severe frostbite injuries. Case Report: The patient, found after six days in freezing conditions, exhibited necrosis on multiple toes. Initial management included stabilization, intravenous fluids, and rewarming. Subsequent necrectomy and amputation revealed exposed metatarsal bones, necessitating a meticulous reconstructive strategy. Bilateral ALT flaps were chosen to preserve walking function, with a staged reconstruction involving multiple operations. The patient’s progress, from inpatient care to outpatient follow-ups, is detailed, emphasizing the challenges and decisions in managing severe frostbite injuries. Results: The surgical intervention utilizing bilateral ALT flaps successfully salvaged the patient’s feet. Throughout the postoperative period, wound care, rehabilitation, and outpatient monitoring contributed to positive outcomes. Despite challenges associated with the patient’s ethnic background and nutritional status, the staged reconstruction facilitated effective healing and functional recovery. The use of ALT flaps provided a reliable solution with minimal donor site morbidity. Conclusion: This case highlights the efficacy of bilateral ALT flap reconstruction in salvaging feet following severe frostbite injury. The successful restoration of foot function underscores the importance of early intervention and tailored reconstructive approaches in frostbite management. Despite patient-specific challenges, including nutritional status and limited healthcare resources, the use of ALT flaps facilitated optimal recovery and functional outcomes. Importantly, this report is unique as it describes a novel case of feet salvage using bilateral ALT flaps in severe frostbite injury, with only one similar case previously reported in the literature. This emphasizes the rarity and significance of this specific surgical approach in frostbite management.

## 1. Introduction

Frostbite is an injury of the skin and underlying tissue caused by freezing, which can lead to soft tissue damage, vascular thrombosis, and amputation. The most common cause is exposure to cold-weather conditions. Regular consumption of alcoholic beverages and tobacco products, vagrancy, psychiatric disorders, inadequate protection during unplanned exposure to low temperatures (soldiers), several medications, and working with equipment that uses CO^2^ and NO^2^ increases the risk for frostbite injury [1]. Additionally, African American ethnicity and O blood group type, as well as possession of the angiotensin-converting enzyme DD allele represent risk factors [2,3]. Frostbite can have a broad range of clinical presentations, from injuries that heal entirely without complications to injuries that require severe limb amputation. Cold skin, numbness, prickling feeling, waxy-looking skin, blistering after rewarming, vasospasm, haemorrhage, and ischaemic perfusion injury (in severe cases) are some of the most common signs [4]. Even if there is no severe tissue loss, patients may experience long-term consequences as a result of their frostbite injury. These include vasomotor diseases, neuropathic and nociceptive pain, and frostbite arthritis. In more severe cases, gangrene can occur, resulting in the amputation of the affected part of the limb. Digits of lower limbs are the most common sites for frostbite, most of them including first-degree injuries, followed by hands, nose, and ear pinna [1,2,5]. Restoring the foot’s functioning is crucial when it comes to foot defects. Although primary closure of the defect, or with a skin transplant, is sometimes the solution, the most common option is treatment with a free flap. Due to the size of the defect and the mechanism of the injury, local flaps are most often inadequate [6]. There are multiple options for the free flap reconstruction of complex soft tissue defects in the foot region, such as a free medial plantar flap, a free groin flap, a free anterolateral thigh flap, or a free anteromedial thigh flap [7,8]. The anterolateral thigh flap (ALT) is one of the best options for reconstruction. The ALT flap was first described by Song et al. [9] and has proved to be one of the best options in reconstruction surgery due to its large skin surface area, long vascular pedicle, perforator anatomy (based on cutaneous vessel perforators from lateral femoral circumflex vessels), minimal donor site morbidity as it is an ideal donor site because of its versatility [10,11]. Considering tissue defects, the ALT flap can be harvested as a cutaneous, fasciocutaneous, musculocutaneous, and adipofascial flap. The anterolateral thigh flap is based on either septocutaneous vessels or musculocutaneous perforators [8,12]. Even though septocutaneous based flap is easier to harvest, it occurs in certain rare cases. The lateral circumflex femoral artery’s descending branch penetrating the vastus lateralis muscle is the most often blood supplier of a musculocutaneous based flap, the perforators of which can be harvested by careful intramuscular dissection [12,13]. The descending branch and its concomitant veins cross obliquely with the nerve to the vastus lateralis muscle within the sulcus surrounded by the vastus lateralis and rectus femoris muscles. The significant topographic features for ALT flap harvesting consist of the line between the anterior superior iliac spine and the superolateral border of the patella, which is equivalent to the aforementioned sulcus [8,12,13]. Vascularized nerves, tensor fasciae latae, and corticocancellous split femur can also be used as a part of the ALT flap to provide necessary reconstruction [14,15]. A sensory flap, whose innervation was thoroughly documented by Ribuffo et al., can be created by the anterior branch of the lateral cutaneous femoris nerve [16]. As the specter of frostbite continues to pose intricate challenges in trauma care, we report a case of a severe frostbite feet injury in a young male refugee from Gambia where decisive surgical intervention became imperative. The only acceptable option for preserving the length of the feet was reconstruction with a free flap. The following freezing case illustrates the nuanced considerations and intricate procedures involved in salvaging the feet through bilateral anterolateral thigh flaps, offering a glimpse into the evolving landscape of reconstructive surgery for severe frostbite injuries.

## 2. Case Report

**Figure 1:**
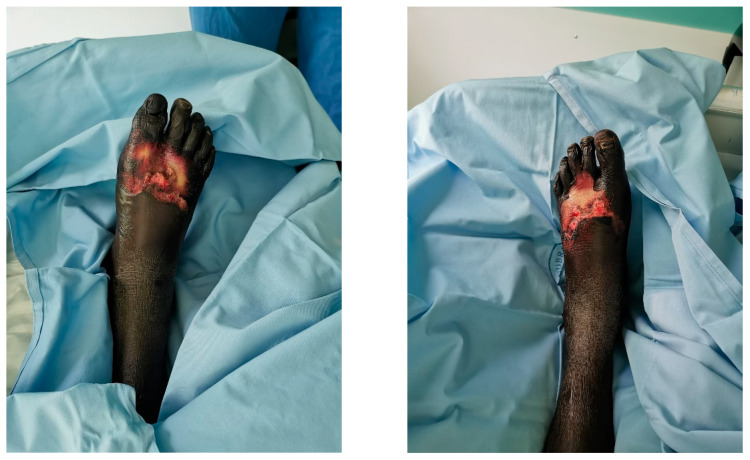
The patient’s feet during admission.

A 19-year-old male refugee from Gambia was brought into the emergency department of our hospital (Figure 1). 

**Figure 2:**
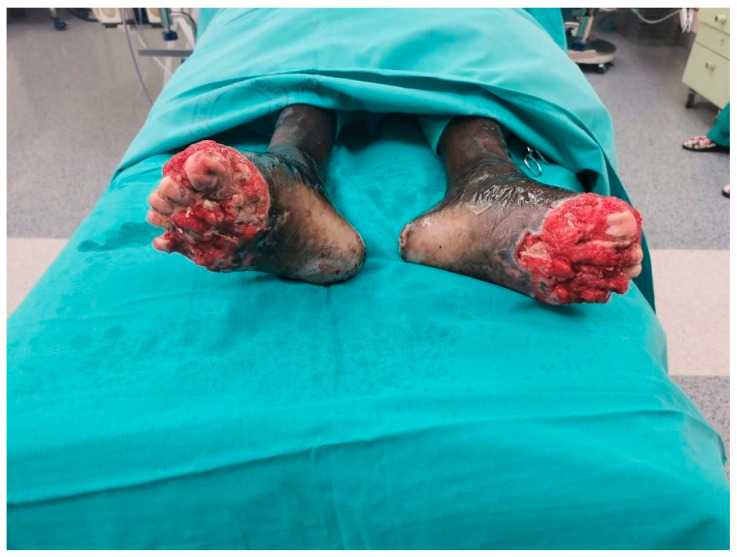
Patient’s feet after five days of negative pressure therapy.

**Figure 3:**
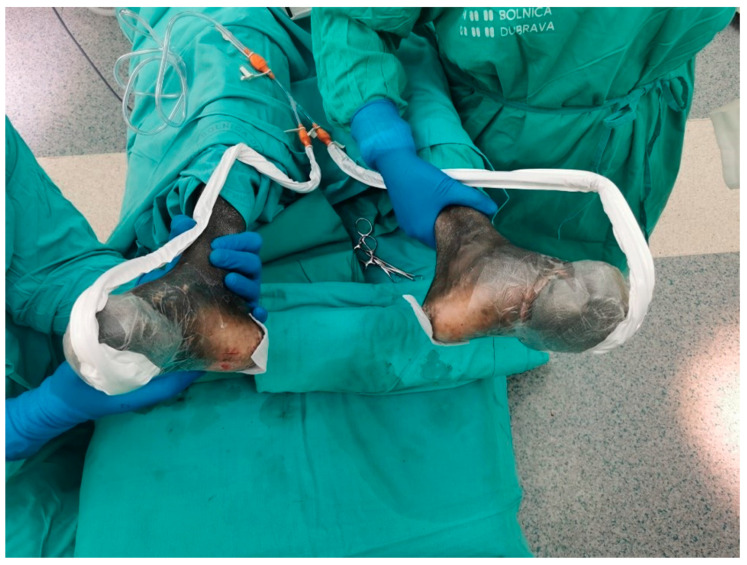
Administration of the NPWT.

Police found him near the highway after he had been wandering for 6 days through the woods. Findings upon initial examinations were: body weight 60 kg, BMI (Body Mass Index) 19.59 kg/m^2^, spO2 99%, HR 102/min, RR 120/80 mmHg, GCS 15, and his axillary temperature was 35.5 °C. A thorough examination by the attending general surgeon showed epidermolysis with partial superficial skin necrosis on the right foot’s first three toes and the left foot’s first toe (fourth-degree frostbite injury). The extent of the frostbites was roughly calculated to be around 4% of the total body surface area. The patient could perform normal movements in both ankle joints. The tibialis posterior and the dorsalis pedis arteries maintained normal flow, as confirmed by Doppler ultrasound. Wound management consisted of wound irrigation and dressings. The patient was extremely dehydrated, undernourished, and felt pain in his feet. Laboratory tests showed a normal red blood cell count, white blood cell count 17.6 × 10^9^/L, neutrophils 14.99 × 10^9^/L, pCO^2^ 5.67 kPa, pO^2^ 11.21 kPa, serum creatinine 62 µM/L, AST (aspartate transferase) 586 U/L, ALT (alanine transaminase) 317 U/L, LD (lactate dehydrogenase) 1146 U/L, creatine kinase 16700 U/L, albumin 24 g/L, total proteins 55 g/L, CRP (C-reactive protein) 158.4 mg/L and myoglobin 1043.6 µg/L. Since the patient did not require emergency surgical treatment, he was admitted to the internal medicine ward. Upon arrival, fluid resuscitation and active external rewarming were started. The elevated laboratory values started to decrease after two days of treatment. Empirical antibiotic therapy was given for 10 days since the patient was considered to have a polymicrobial infection of the skin, soft tissue, and bone, and consisted of meropenem (3 × 1 g IV) and vancomycin (2 × 1 g IV). He also received the Td vaccine and thromboprophylaxis therapy with low-molecular-weight heparin. After the initial treatment was complete, a plastic surgery consultation was requested, and the patient was transferred to the department for plastic and reconstructive surgery. The reconstructive plan consisted of thorough wound cleansing and dressing for two weeks in order to allow complete demarcation of necrotic tissue. Wound swabs and tissue samples for microbiology were taken upon admission to the ward. Microbiology wound cultures revealed coagulase-negative staphylococci, methicillin-resistant Staphylococcus aureus, *Bacillus* sp. and anaerobic Gram-negative bacilli. Afterward, four operations in general anesthesia were performed over 15 days. The first one consisted of the removal of nonviable tissue and amputation in the area of the metatarsophalangeal joints of all toes. Both heads and collumns of all ten metatarsal bones were exposed (Figure 2). Negative pressure wound therapy (NPWT) was applied to the defect on both sides (Figure 3). 

**Figure 4:**
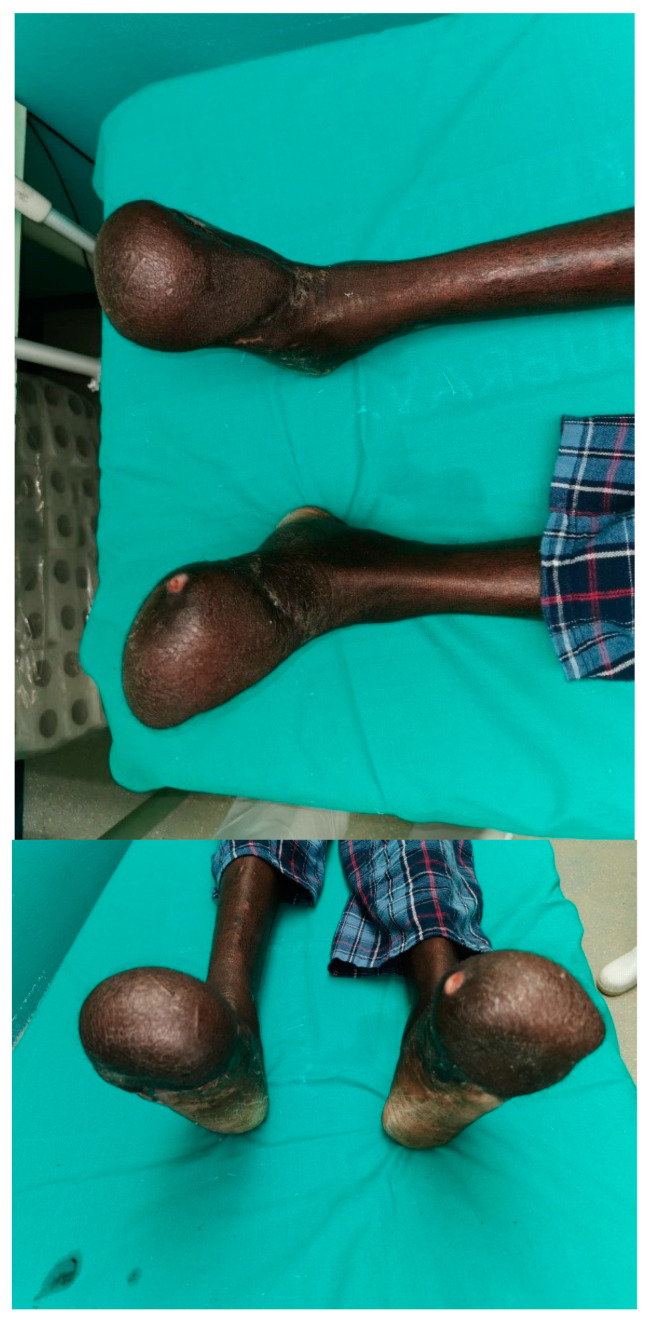
Patient’s feet 3 weeks after the last operation.

Two days later, debridement, and cleaning of both feet were made with reapplication of the NPWT device. The reconstruction of the left foot was performed five days afterwards. A non-innervated fasciocutaneous anterolateral thigh (ALT) flap was harvested from the right upper leg. The patient was placed in a supine position and the AP line was drawn. In the middle of this line, a circle with a radius of 3 cm was drawn, representing the area where the main perforators are most often located. A Doppler probe was used to identify blood vessels. Two musculocutaneous perforators were located. The flap was harvested in a subfascial plane. The exit point of the perforators was shown, and the perforators were dissected through the vastus lateralis muscle in a retrograde manner to their origin from the oblique branch of the lateral circumflex femoral artery. Any encountered small branches were ligated. The ALT flap was designed (17 × 10 × 1 cm) based on the shape and size of the defect. The length of the pedicle was 11 cm since it did not need to be longer because it was anastomosed with the dorsalis pedis artery. On the left foot, the dorsalis pedis artery and vein were found, as well as one surface vein. The stump was debrided, and the ALT flap was placed and molded to create a functionally shaped foot. One artery and two veins were anastomosed. Other small defects on the foot, as well as part of the donor area, were covered with free split-thickness skin transplant (according to Thiersch, 0.2 mm in thickness) harvested by dermatome (AESCULAP^®^ Acculan 4 Dermatome, Tuttlingen, Germany) from the right lower leg. A similar operation was made on the other side one week later. The ALT flap was dissected on the left upper leg. The dimensions of the flap were 18 × 11 × 1.5 cm and the length of the pedicle was 12 cm. Debridement and ostectomy of the right foot with the preparation of the dorsalis pedis artery and concomitant veins were performed. The flap was anastomosed and the donor site was closed primarily. Both reconstruction operations were carried out by two teams working together. One team was harvesting the flap, while the other was preparing the recipient site and performing the anastomosis of the flap vessels to recipient vessels. Two consecutive operations, one week apart, were performed to allow the patient a better recovery and better mobility after the operations. Constant assessment of vitality with a handheld Doppler ultrasound, thermal imaging camera, and capillary refill observation were conducted, as well as regular dressing and care of the flaps. Physical therapy was started on the third postoperative day. Verticalization with walking exercises was allowed on the seventh post-op day. Thirty days after the last operation, the patient was discharged from the hospital. The hospital stay was prolonged because the patient had refugee status. He could not be discharged from the hospital to be managed in an outpatient clinic until all immigration papers were authorized. On discharge, he was able to walk with one crutch and rest with elevated feet. Carbamazepine was prescribed due to phantom pain syndrome, as well as acetylsalicylic acid (2 × 100 mg during one month) to prevent vascular thrombosis. For the next three months, he had regular checkups on an outpatient basis. Wound care consisted of dressings every 3–4 days in a refugee facility care and every 2 weeks as outpatient dressing in our outpatient clinics. Both feet and donor regions healed uneventfully (Figure 4). 

The follow-up period was 3 months. On the last visit, he was able to walk without crutches, and wear normal footwear. The patient was thrilled to be able to walk without crutches and he was very motivated to continue with physical therapy. Unfortunately, a longer patient follow-up was not possible because the patient’s asylum request was refused, and he was deported from the country consequently. We were not able to trace him afterwards.

## 3. Discussion

To determine the best option for the treatment of frostbite, it is necessary to understand the mechanism of its occurrence. The two most important pathophysiological processes are cellular injury and ischemia. Cellular injury is divided into direct and indirect, with the latter causing ischemia. Ice crystal formation, cell dehydration, electrolyte imbalance, and denaturation of lipid-protein complexes are all parts of direct cellular injury that results in cell damage and death [1]. Electrolyte disturbances are caused by the formation of extracellular ice crystals which increase extracellular oncotic pressure and causes cell dehydration [17]. Ischemia is caused by local vasoconstriction after exposure to freezing temperatures. Increased transcapillary plasma loss and oedema formation occur due to increased blood viscosity and microvascular damage caused by cooling of the vascular content. Another cause of ischemia is endothelial damage which leads to the formation of microthrombi and capillary blockage [18]. Frostbite is classified into two or four degrees. The two-tier classification consists of mild and severe, or superficial and deep frostbite injury. The first and second degrees correspond to mild/superficial, while the third and the fourth represent severe/deep frostbite. The depth of tissue freezing, the color of tissues, presence of blistering, necrosis, and oedema differ from first to fourth degree [19,20,21]. Tissues exposed to repetitive freezing and then thawing, frequently exhibit the most serious tissue damage causing thrombosis and ischemia [22]. 

The initial steps to a successful outcome include rewarming and precise fluid (oral or intravenous) resuscitation to prevent further tissue damage. Photo-documenting the appearance of injury at admission and during therapy is advisable to avoid unnecessary dressing removal and allow precise progress analysis [1,21]. Jewelry and other objects that can be restraining must be taken off since swelling will occur during the thawing process. Affected tissue should be elevated and covered in a tight protective dressing with padding between the fingers. If the blister ruptures, to prevent infection and sepsis, it is advised to provide antimicrobials [1,23]. Additionally, tetanus toxoid should be given as a preventive measure. Since rewarming can be extremely painful, a non-steroidal anti-inflammatory drug such as ibuprofen should be given if there are no contraindications [1]. On some occasions, opiates may be needed. Intensive thrombolytic treatment with recombinant tissue plasminogen activator (rtPA) during the first 24 h of frostbite can considerably lessen tissue damage, avert serious morbidity, and save tissue. Iloprost, streptokinase IV, pentoxifylline, and hyperbaric oxygen therapy should all be considered as a part of treatment [1,21,23]. In our case, there were no indications for the aforementioned therapy since more than 48 h had passed since exposure to the cold [24].

Surgical treatment consists of fasciotomy if a compartment syndrome occurs, debridement, amputation (which should be avoided in early stages if possible), negative pressure wound therapy, and tissue reconstruction. It takes about one to three months for nonviable tissue demarcation to be completed, after which reconstructive treatment can be considered [25]. Because of the above mentioned, early debridement is almost contraindicated in nearly all patients since tissue appearing nonviable at first may recover in a few weeks and vice versa. In our case, the reason for not performing a necrectomy during the first two weeks was to make sure the demarcation process of the necrotic tissue was completed. 

The unique morphology and microarchitecture of the plantar area present a challenge in rebuilding the foot’s sole. First, due to its thickness and deformability, the weight-bearing foot can sustain large stresses during compression. The plantar surface’s glabrous skin is made up of a thick epidermal layer that is roughly 1.4 mm thick, as opposed to 0.1 mm in other anatomical areas. Adipose tissue’s many subcutaneous lobules help to absorb and redistribute compressive pressures. Second, a multitude of vertical fibrous septa securely attach the epidermal-dermal junction to the plantar aponeurosis, acting to prevent shearing forces tangential to the skin surface [26,27,28]. The main requirements to perform soft tissue reconstruction are the availability of a solid bone framework, vessels close enough to an injury site capable of sustaining blow flow requirements for the free flap, a stable patient, and a skilled surgeon. The study by Delgado et al. [7] indicates for the first time that axial pattern flaps exhibit a greater integration rate than grafts in the treatment of frostbite. Local and regional flaps improve padding, prevent additional contractures by neovascularization, and allow revascularization of surrounding structures, such as bones and tendons. Free flaps have even more advantages in reconstructive surgery [27]. By using free flaps, it is possible to avoid additional damage to the injured region. A vascularized flap promotes wound healing and decreases the risk of infection. Defect size, function, and aesthetics of recipient and donor sites, dimensions, color, and textures around the defected area, accessible recipient vessels, risk of contamination and the ability to restore sensibility should all be taken into account when choosing a free flap donor site [8]. 

Furthermore, there are not many studies describing the reconstruction of large defects of forefoot. According to a systematic review published by Crowe et al. [26] in 2018, large forefoot defects should be reconstructed with free flaps. 

In our case, two options were considered—amputation for better prosthetic care or covering the feet with free flaps for the purpose of functional feet. Local flaps could not be considered in our case due to defect size and foot tissue microcirculation damage. All patients in whom walking would be preserved without a prosthesis are possible candidates for reconstruction. Even though there were some limiting factors for covering the feet such as a higher risk for severe frostbite injury because of the patient’s African ethnicity [1,18,21] and low serum albumin level which is related to higher in-hospital mortality and nosocomial infection risk, poorer outcome of operation, and longer hospital stay [29,30,31]. Anterolateral thigh flap, gracilis flap, latissimus dorsi flap, and inguinal flap were considered as options for reconstruction. Gracilis flap would be an appropriate choice, but the defect was too large, so we decided against it. The main disadvantage of the latissimus dorsi flap was excessive donor site morbidity. Despite the inguinal flap could cover the defect, it was too thin to achieve the outcome we wanted. We decided to make reconstruction with bilateral anterolateral thigh flaps considering its versatility, accessible location, large amount of soft tissue for coverage, with little donor site morbidity. The reconstruction was even possible to perform in one operating field, which significantly shortened the duration of the procedure. ALT flaps are generally bulky for foot defects reconstruction [27,28]. In our case, ALT flap thickness was much more appropriate since our patient had reduced subcutaneous fat tissue as a result of severe malnutrition. Albumin has essential functions in regulating osmotic pressure, preventing platelet aggregation, influencing the immune system, maintaining physiological pH levels, and wound healing [29,32]. Although fourth-degree bilateral frostbite injury was in favor of amputation, we took the patient’s age into account and performed feet reconstruction to preserve walking function. 

According to the available literature, this is the first case describing feet salvage with bilateral ALT flaps in a man with frostbite injury and trans metatarsal amputations. Young age and refugee status motivated us even more to achieve the best possible result. Only Fodor et al. [33] reported a similar case describing feet salvage with bilateral ALT flaps in a man with a frostbite injury, who spent 8 h in a cold environment. Debridement was combined with bilateral Lisfranc amputations and extended simultaneous innervated ALT flaps were used for reconstruction. The patient had to wear foot orthoses and was able to walk after five years. Our patient spent a much longer time in a cold environment and had African ethnicity (black race) as a risk factor. On the other hand, we did not have to perform Lisfranc amputation. Our aim was to preserve as much length of metatarsal bones as possible to keep the foot as natural as possible and maximize the likelihood of a faster and better gait recovery. Our patient wears trainers instead of foot orthoses and walks after 3 months.

Hong and Kim [6] assessed the outcomes of plantar region coverage with innervated or non-innervated ALT flaps. The innervated group achieved earlier sensitivity recovery, even though the non-innervated developed a protective sensation after 12 months. We did not use the innervated flap to reduce donor morbidity and expected proper reinnervation. Unfortunately, we do not know if the patient developed a protective sensitivity because the patient was deported from the country and a longer follow-up of at least 12 months was not possible. The thinner the flap, the more useful and aesthetically pleasing the outcome, which is the case in our patient. Even though most reconstructions are made after the demarcation line is clearly visible and there is an evident difference between necrotic and viable tissue, according to Lee et al., the optimal early flap reconstruction may be safely accomplished within 10 days after injury without compromising results. Early reconstruction provides the advantage of reduced scarring and fibrosis, which can make flap harvesting more challenging [34]. We did not plan early reconstruction and waited for recovery of as much tissue as possible to achieve better functional results and longer feet.

The lack of research documenting foot reconstruction and the inability to conduct a longer patient’s follow-up are the limiting factors, notwithstanding the importance and severity of this case report.

## 4. Conclusions

In our case, the ALT flap reconstruction of feet after frostbite injury was successful, considering the patient can stand on both legs and walk 3 months after reconstruction. It is important to take into account all positive and negative parameters in order for the patient to accomplish not only the fastest possible recovery, but also the most natural function of the injured area and reduce donor morbidity. To push the uses of this flap to the limit, reconstructive surgeons should be familiar with the versatility of the ALT flap and its possibilities.

## Data Availability

The data used to support the findings of this study are available from the corresponding author upon request.
